# Antibiotic utilization in hospitalized children under 2 years of age with influenza or respiratory syncytial virus infection – a comparative, retrospective analysis

**DOI:** 10.1186/s12879-020-05336-5

**Published:** 2020-08-17

**Authors:** Cihan Papan, Meike Willersinn, Christel Weiß, Michael Karremann, Horst Schroten, Tobias Tenenbaum

**Affiliations:** 1 Pediatric Infectious Diseases, Medical Faculty Mannheim, Heidelberg University, Mannheim, Germany; 2 Center for Infectious Diseases, Institute of Medical Microbiology and Hygiene, Saarland University, Kirrberger Strasse, Building 43, 66421 Homburg, Germany; 3 Institute of Medical Statistics and Biomathematics, Medical Faculty Mannheim, Heidelberg University, Mannheim, Germany

**Keywords:** Influenza virus, Respiratory syncytial virus, Infants, Children, Antimicrobial stewardship

## Abstract

**Background:**

Infections due to Respiratory Syncytial Virus (RSV) and Influenza virus (FLU) are leading causes of hospitalization in young children. Yet, there is little data on factors associated with antibiotic use in these patients.

**Methods:**

We conducted a retrospective, single-center study of all patients below 2 years of age hospitalized between 2014 and 2018. We compared children with RSV infection to children with FLU infection analyzing clinical characteristics and factors contributing to an increased rate of antimicrobial utilization.

**Results:**

RSV infection was diagnosed in 476/573 (83.1%), FLU in 95/573 (16.6%), and RSV-FLU-co-infection in 2/573 (0.3%) patients. Median age was lower for RSV compared to FLU (4 vs. 12 months; *p* < 0.0001). Children with RSV had longer hospitalization (5 vs. 4 days; *p* = 0.0023) and needed oxygen more frequently (314/476 vs. 23/95; *p* < 0.0001) than FLU patients. There was no significant difference in the overall antibiotic utilization between RSV and FLU patients (136/476 vs. 21/95; *p* = 0.2107). Logistic regression analyses revealed that septic appearance on admission (odds ratio [OR] 8.95, 95% confidence interval [CI] 1.5–54.1), acute otitis media (OR 4.5, 95% CI 2.1–9.4), a longer oxygen therapy (OR 1.40; 95% CI 1.13–1.74) and a higher C-reactive protein (CRP) (OR 1.7, 95% CI 1.5–2.0) were significantly associated with antibiotic use in both groups, but not age or pneumonia.

**Conclusions:**

In our cohort, the rate of antibiotic utilization was comparable between RSV and FLU patients, while for both groups distinct clinical presentation and a high CRP value were associated with higher antibiotic use.

## Background

Respiratory tract infections (RTI) are the most common diagnoses in children, especially below the age of 2 years [1]. They substantially contribute to morbidity and mortality worldwide [2]. Furthermore, RTI are associated with large societal impacts [3], including a high health-economic burden [4], caused by a high hospitalization rate among the very young, causing frequent parental absence from work [5]. Moreover, antimicrobial consumption is heavily driven by RTI [6] due to the clinical challenge of distinguishing between viral and bacterial etiologies and the lack of a reliable diagnostic reference standard [7, 8]. Antimicrobial stewardship (AMS) programs are increasingly implemented [9] to counteract this antibiotic overuse which is strongly associated with the upsurge in antimicrobial resistance [10],

The respiratory syncytial virus (RSV) and the Influenza virus (FLU) account for the majority of RTI in infants leading to hospitalization [11, 12]. With the advent of novel point-of-care tests based on nucleic acid amplification, diagnostic accuracy and turnaround time of results have dramatically increased over the past years, both for RSV and FLU [13, 14]. Yet, there is only limited data indicating the potential clinical benefit of rapid viral testing as a tool of AMS in the hospital setting [15]. The overall quality of evidence was generally regarded low, making only for a “weak recommendation” in the latest Guidelines by the Infectious Diseases Society of America and the Society for Healthcare Epidemiology of America for implementing an AMS program [16].

In a recent systematic review with meta-analysis, including both diagnostic accuracy studies and clinical impact studies, no firm conclusion could be made on the clinical impact of viral testing on antibiotic use due to the heterogeneity of the included studies [17], of which only two had included children.

Little is known about the factors that influence antibiotic prescription in patients with a positive RSV or FLU test. We sought to analyze the rate of antibiotic use in RSV and FLU patients and associated clinical and laboratory factors.

## Methods

### Study setting and design

This retrospective, single-center analysis was conducted at the University Children’s Hospital Mannheim, Medical Faculty Mannheim of the Heidelberg University, Germany. The local ethics committee approved of the study (2018-832R-MA). All patients below 2 years of age hospitalized for RSV or FLU infection between the April 2014 and April 2018 were included, thereby covering 4 complete epidemic seasons. Patients were identified by a hospital database search for the corresponding ICD-10 codes (J12.1, J21.0, J20.5, B97.4; J09-J11.-) as the primary diagnosis and, to avoid selection bias, also as secondary diagnosis. RSV or FLU infection were each defined as a positive result from a nasopharyngeal swab either in a rapid point-of-care test (Sofia® Influenza A + B Fluorescent Immunoassay and Sofia® RSV Fluorescent Immunoassay; Quidel, San Diego, California, USA) or in a multiplex PCR (Biofire® Filmarray® Respiratory Panel, Biomérieux, Marcy-l’Étoile, France), which comprises adenovirus; coronaviruses 229E, HKU1, OC43, and NL63; human metapneumovirus; human rhinovirus/enterovirus; influenza A (including substrains) and B; parainfluenza viruses 1 to 4; RSV; *Bordetella pertussis*; *Chlamydophila pneumoniae*; and *Mycoplasma pneumoniae*.

Chart review included present and past medical history, family history, preexisting antibiotic therapy, clinical findings, results of laboratory, microbiological and radiological investigations, data on therapy, need for oxygen, intensive care, and length of stay. Clinical appearance was defined as documented as the physical examination upon admission, including “well”, “ill”, and “septic” appearance. The clinical syndrome was defined according to the discharge diagnosis and/or the documented clinical symptoms on admission, as either upper respiratory tract infection (URTI), bronchitis/bronchiolitis, pneumonia, or fever of unknown origin (FUO).

Case numbers were compared between the seasons to check for imbalances.

We compared children with RSV infection to children with FLU infection. Especially, clinical characteristics and factors contributing to an increased rate of antimicrobial utilization were analysed of RSV and FLU infected children. Furthermore, we defined subgroups of children within each group and compared those treated with antibiotics (RSV+AB+; FLU+AB+) to those who were not treated with antibiotics (RSV+AB-; FLU+AB-), respectively. Antibiotic utilization was defined as any given dose during the hospital stay. We assessed antibiotic therapy in terms of substance and length of therapy.

### Statistical analysis

Statistical analyses were performed in SAS (Statistical Analysis System, North Carolina, USA). Mann-Whitney U test was used for group comparison between RSV and FLU. For multiple comparisons, Kruskal-Wallis test and one-way analysis of variance were applied. Logistic regressions were performed to assess potential risk factors for antibiotic utilization.

## Results

### Demographic data

**Fig. 1:**
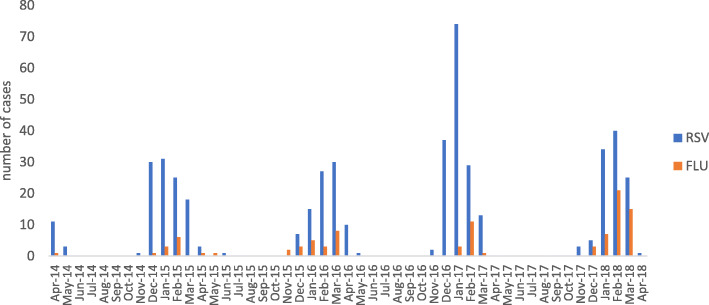
Distribution of cases for each month for RSV (blue) and FLU (orange)

Overall, 573 children were eligible for analysis, with a median age of 5 months (IQR 2–11) and a male predominance of 325/573 (56.7%). Of the 573 children, 476 were identified as having RSV infection (83.1%), and 95 as having FLU infection (16.6%), while in two patients, RSV-FLU co-infection was detected (0.3%). RSV patients were significantly younger than FLU patients (4 vs. 12 months; *p* < 0.0001) (Table 1), while 19.5% of RSV patients were 28 days of age or younger and 47.7% 3 months of age or younger. The majority of RSV patients presented during the months January and February (32.4 and 25.4%), while FLU patients were predominantly diagnosed in February and March (43.2 and 25.3%) (Fig. 1).

**Table 1:** Comparison of clincial and laboratory characteristics of RSV and FLU patients; median and interquartile range are indicated for continuous variables; *ns* not significant

Variables	RSV	FLU	*p*-values
N (%)	476 (83.1%)	95 (16.6%)	
Male	269/476 (56.5%)	55/95 (57.9%)	ns
Age, months	4 (2–8)	12 (5.5–18)	< 0.0001
Duration of illness, days	4 (2–5)	3 (2–5)	0.0005
Fever (≥38.0 °C)	330/476 (69.3%)	93/95 (97.9%)	< 0.0001
Body temperature on admission, °C	37.5 (37.1–38.3)	38.4 (37.5–39.3)	< 0.0001
Peak body temperature, °C	38.7 (37.7–39.4)	39.9 (39.1–40.1)	< 0.0001
Oxygen saturation at admission, %	97 (94–98)	98 (96–100)	0.0002
Minimal oxygen saturation, %	90 (88–94)	96 (90–98)	< 0.0001
Need for intensive care	16/476 (3.4%)	1/95 (1.1%)	ns
Length of stay, days	5 (4–7)	4 (3–6.5)	0.0023
Blood culture obtained	165/476 (34.7%)	54/95 (56.8%)	< 0.0001
CRP on admission, mg/L	6.9 (0–17.6)	6.6 (0–14.1)	ns
Peak CRP, mg/L	12.3 (5.8–29.2)	11.7 (5.4–23.7)	ns
WBC on admission, 10^9/L	10.7 (8.5–13.2)	10.8 (7.1–13.6)	ns
Chest radiography performed	135/476 (28.4%)	18/95 (18.9%)	ns
Infiltrate on chest radiogram	72/135 (53.3%)	13/18 (72.2%)	ns
Preexisting antibiotic therapy	32/476 (6.7%)	14/95 (14.7%)	0.0188
Well appearance	32/476 (6.7%)	6/95 (6.3%)	ns
Ill appearance	245/476 (51.5%)	62/95 (65.3%)	0.0176
Septic appearance	168/476 (35.3%)	23/95 (24.2%)	0.0426
Febrile seizure	3/476 (0.6%)	14/95 (14.7%)	< 0.0001
Acute otitis media	70/476 (14.7%)	33/95 (34.7%)	< 0.0001
Upper respiratory tract infection	0/476 (0%)	47/95 (49.5%)	< 0.0001
Bronchitis/Bronchiolitis	236/476 (49.6%)	6/95 (6.3%)	< 0.0001
Pneumonia	239/476 (50.2%)	21/95 (22.1%)	< 0.0001
Fever of unknown origin	1/476 (0.2%)	21/95 (22.1%)	< 0.0001
Death	0/476	1/95 (1.1%)	ns
Oxygen therapy	314/476 (66.0%)	23/95 (24.2%)	< 0.0001
Length of oxygen therapy, days	2 (0–5)	0	< 0.0001
Antibiotic therapy	136/476 (28.6%)	21/95 (22.1%)	0.2107

### Seasonality

We assessed the seasonal variability (Fig. 1). Numbers were comparable between the seasons except for one season each with a higher burden of RSV (2016/2017) and FLU (2017/2018).

### Baseline risk factors

We assessed patients with respect to a positive past medical history with focus on prematurity, congenital heart disease, congenital diaphragmatic hernia, trisomy 21, and recurrent episodes of bronchitis, but could not find any significant differences, although prematurity and recurrent bronchitis were slightly more common in RSV (4.4 and 4.8%, respectively) than in FLU patients (2.1 and 3.1%, respectively), both missing statistical significance. Furthermore, similar sick contacts were observed for 38.9% of RSV and for 45.3% of FLU patients (statistically not significant).

### Clinical presentation, treatment and outcome of RSV and FLU patients

Both patient groups presented with similar durations of illness, but a significant number of FLU patients had received antibiotics before presenting to the hospital (14.7% vs. 6.7%; *p* = 0.0188). The clinical syndrome diagnosed upon admission was lower respiratory tract infection (49.6%) or pneumonia (50.2%) for most of the RSV patients (compared to 6.3% und 22.1% for FLU patients, respectively; both *p* < 0.0001), while in FLU patients, upper respiratory tract infections (49.5%) and FUO (22.1%) accounted for the majority of clinical manifestations (compared to 0.2 and 0% for RSV patients, respectively; both *p* < 0.0001). In association with this, fever, and also febrile convulsions were significantly more often found in FLU patients (Table 1). Acute otitis media was significantly more often diagnosed among FLU patients (*p* < 0.0001).

RSV patients needed significantly more often oxygen therapy (66% vs. 24.2%; *p* < 0.0001) and salbutamol (68.3% vs. 27.1%; *p* < 0.0001) than FLU patients, and had longer hospital stays (5 vs. 4 days; *p* = 0.0023), while there were no statistically significant differences concerning the need for intensive care and antibiotic therapy. One FLU patient died due to severe pneumonia with suspected bacterial co-infection, leading to respiratory failure.

### Laboratory, microbiological and radiological findings in RSV and FLU patients

No significant differences were found between both groups concerning C-reactive protein (CRP) and white blood cell counts (WBC). Chest radiographs were performed slightly more often in RSV patients (28.4% vs. 19.8%; not significant), while infiltrates were slightly more frequently diagnosed among FLU patients (72.2% vs. 53.3%; not significant).

For 19 patients, a multiplex PCR on a nasopharyngeal swab was available (14 RSV patients, 5 FLU patients). Among the 14 patients with RSV infection for whom a multiplex PCR was available, adenovirus was concomitantly detected in two patients and coronavirus and *Bordetella pertussis* in one patient, each. In FLU patients, adenovirus and human metapneumovirus were detected in one patient, each. Blood cultures were ordered in 165 patients with RSV, of whom 9 were positive, all of which were deemed contaminants (5 coagulase negative staphylococci; 1 co-detection of *Staphylococcus epidermidis*, *Streptococcus mitis*, and *Streptococcus peroris*; 1 co-detection of *Micrococcus luteus* and *Pseudomonas stutzeri*; 1 *Micrococcus luteus*; 1 *Rothia dentocariosa*). For FLU patients, blood cultures were ordered in 54, with 3 positive cultures, all regarded as contamination (2 *Staphylococcus hominis*; 1 *Corynebacterium afermentans*).

### Antibiotic utilization

**Fig. 2:**
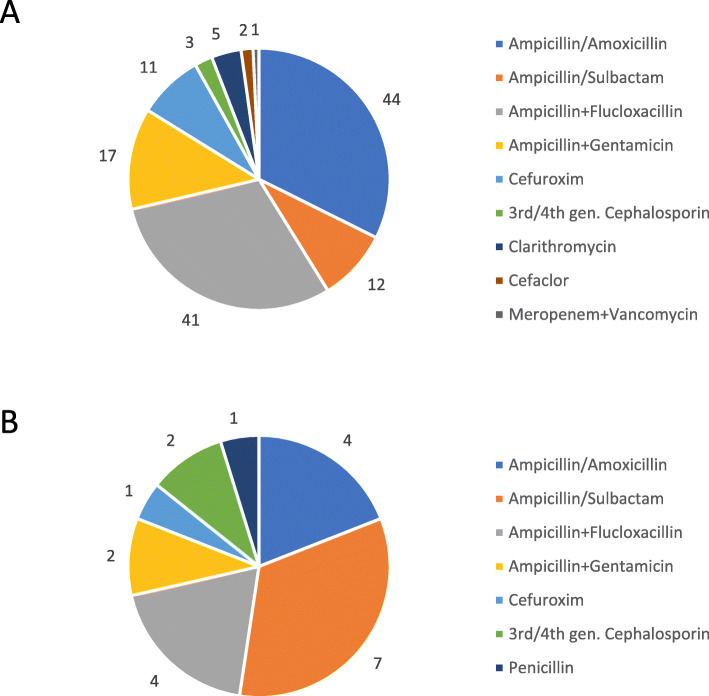
Distribution of empiric antibiotics prescribed after hospitalization, absolute numbers; **a** RSV patients (*N* = 136); **b** influenza patients (*N* = 21); gen.: generation

Antibiotics were prescribed in 136/476 (28.6%) of RSV patients and 21/95 (22.1%) of FLU patients (*p* = 0.2107). A monotherapy was used in 77/136 (56.6%) in RSV patients and 15/21 (71.4%) of FLU patients (*p* = 0.2395), while the remaining fractions were prescribed a combination therapy. For RSV patients, the most frequently ordered therapies were aminopenicillin monotherapy in 44/136 (32.4%), ampicillin + flucloxacillin combination therapy in 41/136 (30.1%), and ampicillin + gentamicin combination therapy in 17/136 (12.5%). For FLU patients, ampicillin/sulbactam was prescribed in 7/21 (33.3%), followed by an aminopenicillin monotherapy in 4/21 (19.0%) and ampicillin + flucloxacillin combination therapy in 4/21 (19.0%). The distribution of prescribed antibiotics is depicted in Fig. 2. The median length of antibiotic therapy was 5.5 days for RSV patients (IQR 4–7) and 6 days for FLU patients (IQR 4–8) (*p* = 0.3680).

### Subgroup comparison – RSV patients

We compared RSV patients who received antibiotics (RSV+AB+) during the hospital stay to those without antibiotics (RSV+AB-). Children who were RSV+AB+ were significantly older than RSV+AB- (6 vs. 3 months; *p* = 0.0025) (Table 2). Furthermore, they presented significantly more often with fever (91.2% vs. 60.6%; *p* < 0.0001), a septic appearance (48.5% vs. 30%; *p* = 0.0002), pneumonia (76.8% vs. 39.1%; *p* < 0.0001), acute otitis media (26.5% vs. 10%; *p* < 0.0001), and a higher CRP on admission (26.9 mg/L vs. 4.3 mg/L; *p* < 0.0001). During the hospital stay, RSV + AB+ children had significantly higher rates of chest radiographies (60.3% vs. 15.6%; *p* < 0.0001), blood cultures obtained (52.2% vs. 27.6%; *p* < 0.0001), oxygen therapy (80.1% vs. 60%; *p* < 0.0001), intensive care stays (9.6% vs. 0.9%; *p* < 0.0001), and longer hospital stays in total (6 days vs. 5 days; *p* < 0.0001).

**Table 2:** Comparison of clincial and laboratory characteristics of RSV and FLU patients receiving antibiotics (AB+) to those not receiving antibiotics (AB-); median and interquartile range are indicated for continuous variables

Variables	RSV AB+	RSV AB-	*p*-values	FLU AB+	FLU AB-	*p*-values
N (%)	136/476 (28.6%)	340/476 (71.4%)		21/95 (22.1%)	74/95 (77.9%)	
Male	76/136 (55.1%)	193/340 (56.8%)	ns	11/21 (52.4%)	44/74 (59.5%)	ns
Age, months	6 (2–12)	3 (2–7)	0.0025	14 (9–18)	11 (5–17.8)	ns
Duration of illness, days	4 (2–5)	3 (2–5)	ns	3 (2–5.3)	2 (2–4)	ns
Fever (≥38.0 °C)	124/136 (91.2%)	206/340 (60.6%)	< 0.0001	20/21 (95.2%)	73/74 (98.6%)	ns
Body temperature on admission, °C	38.0 (37.4–38.8)	37.4 (37.0–38.1)	< 0.0001	38.2 (37.7–39-4)	38.4 (37.4–39.3)	ns
Peak body temperature, °C	39.2 (38.8–39.8)	38.2 (37.5–39.2)	< 0.0001	40.0 (39.4–40.1)	39.8 (39.1–40.0)	ns
Oxygen saturation at admission, %	96 (93–98)	97 (95–98)	0.0019	97 (93.3–98.3)	98 (97–100)	ns
Minimal oxygen saturation, %	89 (88–92)	91 (88–95)	< 0.0001	90 (87.8–95.3)	96 (95–98)	ns
Need for intensive care	13/136 (9.6%)	3/340 (0.9%)	< 0.0001	1/21 (4.8%)	0/74	ns
Length of stay, days	6 (5–8.3)	5 (3–7)	< 0.0001	6 (5–8.5)	5 (3–7)	0.0003
Blood culture obtained	71/136 (52.2%)	94/340 (27.6%)	< 0.0001	12/21 (57.1%)	42/74 (56.8%)	ns
CRP on admission, mg/L	26.9 (9.7–51.2)	4.3 (0–10.6)	< 0.0001	23.1 (5.9–58.4)	4.9 (0–13.0)	< 0.0001
Peak CRP, mg/L	40.5 (19.2–61.5)	7.9 (4.5–14.4)	< 0.0001	54.9 (11.7–76.8)	9.4 (4.2–14.2)	< 0.0001
WBC on admission, 10^9^/L	11.6 (8.5–15.2)	10.5 (8.5–12.7)	ns	9.9 (7.6–12.0)	10.9 (7.1–13.7)	ns
Chest radiography performed	82/136 (60.3%)	53/340 (15.6%)	< 0.0001	11/21 (52.4%)	7/74 (9.5%)	< 0.0001
Infiltrate on chest radiogram	48/82 (58.5%)	24/53 (45.3%)	ns	8/11 (72.7%)	5/7 (71.4%)	ns
Preexisting antibiotic therapy	19/136 (14.0%)	13/340 (3.8%)	0.0002	6/21 (13.7%)	8/74 (3.9%)	ns
Well appearance	1/136 (0.7%)	31/340 (9.1%)	0.0004	2/21 (9.5%)	4/74 (5.4%)	ns
Ill appearance	60/136 (44.1%)	185/340 (54.4%)	0.0536	12/21 (57.1%)	50/74 (67.6%)	ns
Septic appearance	66/136 (48.5%)	102/340 (30%)	0.0002	6/21 (28.6%)	17/74 (23.0%)	ns
Acute otitis media	36/136 (26.5%)	34/340 (10%)	< 0.0001	12/21 (57.1%)	21/74 (28.4%)	0.0199
Upper respiratory tract infection	0/136 (0%)	0/340 (0%)	ns	5/21 (23.8%)	42/74 (56.8%)	0.0123
Bronchitis/Bronchiolitis	31/136 (22.5%)	207/340 (60.9%)	< 0.0001	1/21 (4.8%)	5/74 (6.8%)	ns
Pneumonia	106/136 (76.8%)	133/340 (39.1%)	< 0.0001	11/21 (52.4%)	10/74 (13.5%)	0.0005
Fever of unknown origin	0/136 (0%)	0/340 (0%)	ns	4/21 (19.0%)	17/74 (23.0%)	ns
Oxygen therapy	109/136 (80.1%)	204/340 (60%)	< 0.0001	12/21 (57.1%)	11/74 (14.9%)	0.0002
Length of oxygen therapy, days	3 (2–6)	2 (0–4)	< 0.0001	1 (0–6)	0	0.0002

### Subgroup comparison – FLU patients

For FLU patients, a significant difference upon clinical presentation was a higher rate of pneumonia (52.4% vs. 13.5%; *p* = 0.0005) and acute otitis media (57.1% vs. 28.4%; *p* = 0.0199) among children receiving antibiotics (FLU+AB+) compared to children without antibiotics (FLU+AB-) (Table 2). Apart from that, FLU+AB+ children had significantly higher CRP on admission (23.1 mg/L vs. 4.9 mg/L; *p* < 0.0001) and peak CRP (54.9 mg/L vs. 9.4 mg/L; *p* < 0.0001) in the course of disease; a higher rate of chest radiography performed (52.4% vs. 9.5%; *p* < 0.0001), higher need for oxygen therapy (57.1% vs. 14.9%; *p* = 0.0002), and a longer hospital stay (6 days vs. 5 days; *p* = 0.0003).

### Risk factors for antibiotic use

We performed a multivariate logistic regression to identify risk factors associated with antibiotic utilization among both RSV and FLU. The best model fit for antibiotic use at any time point was achieved for the following factors (c-index 0.916) (Table 3): otitis media (odds ratio 8.33; 95% confidence interval 3.58–19.37), peak CRP (OR 1.77; 95% CI 1.54–2.04), and the length of oxygen therapy (OR 1.40; 95% CI 1.13–1.74). Septic appearance (OR 4.882; 95% CI 0.866–27.505; *p* = 0.0723) was statistically not significant.

**Table 3:** Logistic regression model for antibiotic use at any time point

Variables	Odds ratio	95% confidence interval	*p*-value
Otitis media	8.325	3.577–19.374	< 0.0001
Peak CRP	1.772	1.538–2.041	< 0.0001
Length of oxygen therapy	1.404	1.130–1.744	0.0022
Septic appearance	4.882	0.866–27.505	0.0723

The best model fit for antibiotic use on admission (c-index 0.896) (Table 4) was achieved for otitis media (OR 4.5, 95% CI 2.1–9.4), CRP on admission (OR 1.7, 95% CI 1.5–2.0), and septic appearance (OR 8.95, 95% CI 1.5–54.1). Pneumonia as a potential risk factor (OR 3.629, 95% CI 0.902–14.602) missed statistical significance.

**Table 4:** Logistic regression model for antibiotic use on admission

Variable	Odds ratio	95% confidence interval	*p*-value
Otitis media	4.496	2.141–9.439	< 0.0001
CRP on admission	1.687	1.457–1.953	< 0.0001
Septic appearance	8.950	1.481–54.087	0.0169
Pneumonia	3.629	0.902–14.602	0.0696

## Discussion

In this large retrospective four-season-analysis we present data on more than 570 children below the age of 2 years with RSV or FLU infection, demonstrating a high antibiotic use in a substantial proportion of children.

Recent systematic reviews with meta-analyses had shown that viral testing, including FLU and RSV, does not affect antibiotic prescription rates [17, 18], indicating that other factors pertaining to the decision-making of physicians need to be addressed by AMS measures.

Although RSV patients were significantly younger in our cohort, this apparently did not contribute to a higher rate of antibiotic utilization, as RSV children with antibiotics were significantly older than RSV children that did not receive antibiotics. Instead, driving factors for a potential antibiotic overuse were a high CRP, the presence of acute otitis media, septic appearance and the length of oxygen therapy.

FLU patients presented significantly more often with fever, febrile convulsions, acute otitis media, and upper respiratory tract infection or fever of unknown origin, while RSV patients presented significantly more often with a lower oxygen saturation, septic appearance, and lower respiratory tract infection or pneumonia.

We found higher rates of blood cultures obtained from FLU patients than from RSV patients, which in large part can be explained by the higher rate of febrile children in the FLU cohort, while chest radiographies were more frequently performed among those who received antibiotics within both groups. Interestingly, we found rates of chest infiltrates comparable between treated and non-treated patient groups. One possible explanation is that performing a chest radiograph serves as a surrogate for disease severity and hence influences the decision to prescribe antibiotics. In previous studies, it was hypothesized that less specific radiologic findings may have pushed physicians towards antibiotic treatment, even in the absence of infiltrates [19]. Still, it is generally advised against routinely performing chest radiographs in children who present with typical clinical findings of viral bronchiolitis, of which the majority is caused by RSV [20].

Male children were more predominant in both RSV and FLU cohorts, which had been reported similarly in previous studies [21], yet without convincing explanations so far [22]. Of note, the rate of antibiotic use before admission was 2.2 times higher among FLU patients, while during hospitalization, more RSV patients received antibiotics.

The antibiotic utilization in 28.6% of RSV and 22.1% of FLU patients respectively is lower than in comparable cohorts published in the recent past [23, 24], which may reflect a stronger reluctance to use antibiotics in children with point-of-care test confirmed viral infections at our hospital.

Complex medical comorbidities, such as chronic lung disease due to prematurity and congenital heart disease, are known risk factors for hospitalization due to RSV infection [25, 26]. In our cohort, we did not find any difference in these comorbidities between RSV and FLU patients.

The strengths of our study are the study size, allowing for robust statistical analyses, the long inclusion period, accounting for fluctuations of virus seasonality, and the consistency in standard of care during the inclusion period, thereby avoiding unwanted biasing effects.

Certain limitations of the study also merit critical appraisal. First, we obtained data retrospectively, bringing about the usual constraints and biases inherent to the nature of retrospective analyses. As such, we could not assess the effect of vaccination coverage, especially pertaining to influenza, on the study outcomes since these data were not systematically obtained. In Germany, currently no general recommendation for FLU vaccination for children exists, but only for those with chronic medical conditions. Secondly, no reference method was applied with regard to the correctness of antibiotic utilization. The correct judgement whether an antibiotic is warranted or not is hindered by the lack of a reference standard. Several methods exist to account for this, e.g. a panel of expert referees, blinded to each other, reviewing each case and adjudicating on the “correct” etiology, i.e. bacterial, viral, bacterial-viral co-infection, or unknown, as applied in other studies [21, 27]. Third, our cohort lacked an additional systematic viral testing beyond RSV and Influenza for all patients, making it impossible to judge whether other viruses, such as adenovirus, may have played a causative role, especially in cases with higher CRP [28]. Other viruses, albeit rarer, such as the human metapneumovirus, can be equally or even more associated with antibiotic overuse in children, as described by Schreiner and colleagues [29]. An extensive pathogen testing in conjunction with a control group of healthy children may have helped to better appraise the role of other viruses or bacteria, since some microorganisms can be found abundantly also in healthy controls [11], and the co-presence of some pathogens have been reported to correlate with disease severity [30]. Of note, O’Grady and colleagues had found that more than half of children with an acute respiratory infection had a viral-bacterial “co-detection” in nasal swabs, the bacterium in most cases being *Haemophilus influenzae* [31]. In another study in children with lower respiratory tract infections [32], the most common detected potentially pathogenic colonizers were *Haemophilus influenzae* (32.1%), *Moraxella catharralis* (26.7%), *Staphylococcus aureus* (17.7%) and *Streptococcus pneumoniae* (16.7%). Nevertheless, detection of bacterial colonizers may not help in the decision to initiate antibiotic therapy. Instead, there is evidence that the utilization of viral point-of-care diagnostics may prevent the usage of antibiotics [33, 34]. Overall, this fits well into the concept of diagnostic as well as antibiotic stewardship. Fourth, our cohort had a low case fatality rate, which reduces comparability to cohorts from other backgrounds, e.g. from low- and middle-income countries, where mortality may be higher [35]. Finally, the low baseline rate of antibiotic utilization may limit the generalizability of our results to other settings where the culture of prescribing antibiotics especially in infants and young children below the age of 2 years may differ [36] due to a lack of AMS or other reasons.

In light of the imperfection of both clinical features [7] and laboratory parameters such as CRP [37, 38], novel diagnostics measures are needed to better distinguish between viral and bacterial infections and thereby help reducing unwarranted antibiotic therapy. New host-protein based assays, e.g. combining CRP with Myxovirus resistance protein A [39], but also combinations of CRP and tumor-necrosis-factor related apoptosis inducing ligand and interferon-gamma induced protein 10, have shown very convincing results in studies [21, 40–42], with high diagnostic accuracy and a potential to dampen unnecessary antibiotic use. Transcriptomic signatures have also shown to be very accurate in establishing the true etiology [43, 44], and although cost issues and turnaround times steadily improve [45], practical feasibility and implementation into the clinical workflow are persisting challenges still to be taken.

## Conclusions

In summary, we show that clinical appearance on admission, the presence of otitis media, but also a high CRP and the length of oxygen therapy are the main factors associated with antibiotic use in our cohort of in children below the age of 2 years with RSV or FLU infection. It remains to be proven in prospective studies if AMS interventions aiming at viral testing, e.g. as multiplex PCR, in conjunction with more precise biomarkers or combinations thereof can help to effectively reduce antibiotic treatment in children with RSV infection and Influenza.

## Data Availability

The datasets used and analysed during the current study are available from the corresponding author on reasonable request.

## Abbreviations

AB: Antibiotic
AMS: Antimicrobial stewardship
CRP: C-reactive protein
FLU: Influenza virus
FUO: Fever of unknown origin
IQR: Interquartile range
OR: Odds ratio
PCR: Polymerase chain reaction
RSV: Respiratory syncytial virus
RTI: Respiratory tract infection
URTI: Upper respiratory tract infection
WBC: White blood cell count
